# Successful treatment of *Stenotrophomonas maltophilia* meningitis in a preterm baby boy: a case report

**DOI:** 10.4076/1752-1947-3-7389

**Published:** 2009-07-17

**Authors:** Pilar Rojas, Elisa Garcia, Gema M Calderón, Fernando Ferreira, Marisa Rosso

**Affiliations:** 1 Neonatology Unit, Virgen del Rocío University Children's Hospital, Av. Manuel Siurot s/n, 41013 Seville, Spain

## Abstract

**Introduction:**

*Stenotrophomonas maltophilia* is an important cause of hospital acquired infection particularly among severely debilitated and immunosuppressed patients.

**Case presentation:**

We report a case of *S. maltophilia* meningitis in a preterm baby boy after a neurosurgical procedure, successfully treated with trimethoprim-sulfamethoxazole and ciprofloxacin.

**Conclusion:**

This organism should be considered as a potential cause of meningitis and trimethoprim-sulfamethoxazole and ciprofloxacin are a combination that is successful and safe for treating preterm infants.

## Introduction

*Stenotrophomonas maltophilia* (*S. maltophilia*) is a non-fermentative Gram-negative bacillus, previously known as *Pseudomonas maltophilia* and later *Xanthomonas maltophilia*. This bacterium is found in several environments such as water, soil, plants, food and hospital settings [1,2]. It is increasingly recognised as a significant cause of hospital acquired infection particularly among severely debilitated and immunosuppressed patients, those receiving long-term antimicrobial therapy and those with indwelling central venous catheters. The resultant infections are extensive, with the respiratory tract, soft tissues and the skin most frequently involved [1]-[4]. Although the pathogen is considered to be an infrequent cause of meningitis, it has become a focus of interest not only due to increasing recognition of its pathogenic potential but also because of its marked antibiotic resistance [1].

Here we report a case of *S. maltophilia* meningitis in a baby boy after a neurosurgical procedure, successfully treated with the combination of trimethoprim-sulfamethoxazole and ciprofloxacin.

## Case presentation

A baby boy was delivered at 26 weeks of gestation after a spontaneous rupture of membranes. The patient was admitted to our neonatal intensive care unit with Apgar scores of two and eight at 1 and 5 minutes, respectively. On the 12th day of his life clinical and radiological signs of perforated necrotizing enterocolitis (NEC) occurred and required surgical intestinal resection. Cerebral ultrasound at day 15 of his life was performed and showed intraventricular haemorrhage and dilated cerebral ventricles. Because of NEC, temporary external cerebrospinal fluid (CSF) drainage was inserted. Two weeks after external CSF drainage was performed, he developed *Klebsiella BLEE* meningitis. Antibiotic therapy with meropenem was started and the external CSF drainage was replaced. After 19 days of treatment with meropenem a new CSF sample from drainage revealed 1200 cells/mm^3^ (95% neutrophils), protein 3.4 g/L and glucose 0.03 g/L. Gram-negative bacillus were seen on gram strain in the CSF culture and it was positive for *S. maltophilia*. The strain was only susceptible in vitro to trimethoprim-sulfamethoxazole (TMP-SMX), with a mean inhibitory concentration (MIC) of ≤2/38, minocycline and ciprofloxacin. TMP-SMX intravenous therapy (50 mg/kg per day in two divided doses) was commenced. The external ventricular drainage was not removed at this stage because the patient's state was critical. The next sample analysis of CSF from the drainage 14 days after starting TMP-SMX revealed the following profile: white blood cell count of 1300 cells/mm^3^ (90% neutrophils), protein 1.39 g/L and glucose 0.04 g/L. The CSF culture was still positive for *S. maltophilia* and consequently ciprofloxacin (15 mg/kg per day in two divided doses) was added to TMP-SMX. Furthermore, the external ventricular drainage was removed and after 7 days of therapy with ciprofloxacin in combination with TMP-SMX, the analysis of the CSF was normal and the culture was sterile. Finally, 21 more days of therapy were completed with both antibiotics. No adverse effects were found during ciprofloxacin treatment. There was no displacement of bilirubin with the use of sulfamethoxazole in our patient and the values were normal (maximum total bilirubin 1.27 mg/dL). A ventricular-peritoneal shunt was inserted after the infection was eradicated due to severe ventricular dilatation.

## Discussion

*S. maltophilia* is an extremely rare cause of meningitis and, to our knowledge, only 15 cases, including our case, have been reported to date [2]. Four cases were children including two neonates with *S. maltophilia* meningitis. The median age for the two female and two male infants was 5.3 months; range from 4 days to 13 months [2]-[5]. The two previous patients with neonatal *S. maltophilia* meningitis were preterm infants and were 4 and 7 days old, respectively. One patient died immediately after presentation and the other one was successfully treated with ciprofloxacin because of multi-resistant *S. maltophilia* (Table 1).

**Table 1:** Details of children with meningitis caused by *S. maltophilia*

Case No	Reference/Year	Age/sex	Neurosurgical procedure	Therapy	Outcome
1	(1) 1977	8 months/male	None	Ampicilin, colistin	Died
2	(1) 1977	13 months/ female	None	Chloramphenicol, sulphadoxine	Recovered
3	(2) 1984	7 days/male	None	None	Died
4	(3) 2002	4 days/female	None	Ciprofloxacin	Recovered
5	Present report	69 days/male	External (CSF) drainage	TMP-SMX, ciprofloxacin	Recovered

The reported risk factors associated with *S. maltophilia* infection are prematurity, neurosurgical procedures (especially shunts and drainages), intracranial haemorrhages and malignancies [6]. Our patient had undergone several neurosurgical procedures and also, importantly, had been treated with a previous broad-spectrum antibiotic such as carbapenem, which is also a suggested risk factor for infection with *S. maltophilia*[1,6].

*S. maltophilia* is increasingly recognised as a cause of nosocomial infections of special interest because of its intrinsic resistance to multiple antimicrobial agents used to treat Gram-negative infections. It is resistant to a variety of antibiotics, for example aminoglycosides, ß-lactam agents and it is intrinsically resistant to carbapenems. Based on susceptibility studies, TMP-SMX is the drug of choice for treatment of *S. maltophilia* infections. However, recent data indicate that the percentage of strains resistant to TMP-SMX may be increasing [1,3,7,8]. In this patient the pathogen was susceptible to this antimicrobial therapy but CSF cultures only became sterile after removal of the external ventricular drainage and the addition of ciprofloxacin to TMP-SMX. We decided to add ciprofloxacin because TMP-SMX is bacteriostatic and the infant was seriously ill. The administration of sulfamethoxazole, which binds to albumin and competes with bilirubin, can increase the possibility of hyperbilirubinaemia and serious neurological complications such as kernicterus in neonates. This was not observed in our patient.

With respect to ciprofloxacin, the optimal dose and duration of the treatment for neonatal Gram-negative meningitis remains uncertain. Lo et al. reported a preterm infant with multi-resistant *S. maltophilia* (including resistance to TMP-SMX) meningitis successfully treated with ciprofloxacin [3]. Due to the small number of cases and the short follow-up periods, further studies are needed to establish dosage, CSF ciprofloxacin concentrations and duration of treatment for meningitis. There is also very little information available on this drug sequelae [3,7,8]. The severe ventricular dilatation in our patient was due to intraventricular haemorrhage and the hearing deficit noted could be a consequence of meningitis. Long-term follow-up, including routine neurologic examination as well as visual and auditory evoked potentials, is obligatory. Our patient is the first case of *S. maltophilia* meningitis in a preterm infant successfully treated with the antimicrobial combination of TMP-SMX and ciprofloxacin.

## Conclusions

Due to the increase in meningitis caused by *S. maltophilia* in neurosurgical cases, and its marked resistance to antibiotics normally used to treat Gram-negative nosocomial infections, it should be considered as a potential cause of meningitis in patients with external ventricular drainage and long-term broad-spectrum antimicrobial therapy.

## Abbreviations

CSF: cerebrospinal fluid; MIC: mean inhibitory concentration; NEC: necrotizing enterocolitis; TMP-SMX: trimethoprim-sulfamethoxazole.

## Consent

Written informed consent was obtained from the patient's mother for publication of this case report and any accompanying images. A copy of the written consent is available for review by the Editor-in-Chief of this journal.

## Competing interests

The authors declare that they have no competing interests.

## Authors' contributions

PR made substantial contributions to conception, design, acquisition of data, analysis and interpretation of data and drafting the manuscript. EG acquired and analysed the data. GMC contributed to the analysis and interpretation of the data. FF helped with the acquisition of the data. MR was involved in drafting the manuscript, revising it critically for important intellectual content and final approval of the version. All authors read and approved the final manuscript.

## References

[B1] Nicodemo AC García Paez JI Antimicrobial therapy for Stenotrophomonas maltophilia infections Eur J Clin Microbiol Infect Dis 2007 26 229 237 10.1007/s10096-007-0279-3 17334747

[B2] Yemisen M Mete B Tunali Y Yentur E Ozturk R A meningitis case due to Stenotrophomonas maltophilia and review of the literature Int J Infect Dis 2008 23 1 3 18579427 10.1016/j.ijid.2008.03.028

[B3] Wen-Tsung Lo Chin-Chien Wang Chuen-Ming Lee Mong-Ling Chu Successful treatment of multi-resistant Stenotrophomonas maltophilia meningitis with ciprofloxacin in a pre-term infant Eur J Pediatr 2002 161 680 682 10.1007/s00431-002-1095-5 12536991

[B4] Denis F Sow A David M Etude de deux cas de meningitis a Pseudomonas maltophilia observes au Senegal Bull Soc Med Afr Noire Lang Fr 1977 22 135 139 589735

[B5] Sarvamangala Devi JN Venkatesh A Shivananda PG Neonatal infections due to Pseudomonas maltophilia Indian Pediatr 1984 21 72 74 6698592

[B6] Caylan R Aydin K Koksal I Meningitis cause by Stenotrophomonas maltophilia: case report and review of the literature Ann Saudi Med 2002 22 216 218 17159399 10.5144/0256-4947.2002.216

[B7] Krcmery V Jr Filka J Uher J Kurak H Sagat T Tuharsky J Novak I Urbanova T Kralinsky K Mateicka F Krcméryová T Jurga L Sulcová M Stencl J Krúpová I Ciprofloxacin in treatment of nosocomial meningitis in neonates and in infants: report of 12 cases and review Diagn Microbiol Infect Dis 1999 35 75 80 10.1016/S0732-8893(99)00052-8 10529884

[B8] Van den Oever HL Versteegh FG Thewessen EA van den Anker JN Mounton JW Neijens HJ Ciprofloxacin in preterm neonates: case report and review of the literature Eur J Pediatr 1998 157 843 845 10.1007/s004310050949 9809826

